# “Rejoice Therapy”: Creating and Shaping 'Joy' according to St. Paul of Tarsus

**DOI:** 10.1007/s10943-023-01839-y

**Published:** 2023-06-12

**Authors:** Dariusz Iwański

**Affiliations:** Faculty of Theology, Wydział Teologiczny UMK, Nicolaus Copernicus University, ul. Gagarina 37, 87-100 Toruń, Poland

**Keywords:** Joy, Rejoice therapy, Shaping emotions, Rejoice always, Creating joy, St. Paul of Tarsus, 1 Thess. 5:16

## Abstract

St. Paul of Tarsus, in 1 Thessalonians (5:16), urges his suffering addressees to ‘rejoice always.’ This can seem not only inappropriate, but even inhumane. It can be argued, however, that a unique therapy to strengthen the disheartened is at work. St. Paul applies to his readers what can be described as an authorial therapeutic method—“rejoice therapy”—to help them create and shape their joy despite the difficult situation in which they live. St. Paul employs more than just rhetorical strategies in order to achieve this intended effect. St. Paul provides his readers with practical and universalistic techniques, which can continue to have therapeutic value today.

## Introduction

“Rejoice always!” is one of the startling pieces of advice St. Paul (c. 4 BCE—c. 64 CE) gives when writing to the Christians in Thessalonica (1 Thess. 5:16). It can be a very disturbing line because the Apostle does not mean it as a pious wish or a polite greeting. It is rather a call for action, a task to be accomplished. A careful reading of the pericope, of which this exhortation is a part, allows one to see that there is more to it than a simple command. St. Paul applies it as a kind of authorial “therapy” to help the recipients overcome an all-pervasive malaise under which they were living. I call it “rejoice therapy.”

But the question remains: How can one demand a constant joyful disposition from someone? Experience tells us that joy, like other emotions, is rather fleeting. If one further considers the situation of the Thessalonians, who are mourning the loss of some members of the community, one might even suspect Paul of exhibiting the complete unrealism inherent in some contemporary motivational speakers. While Paul’s strategy of shaping joy is indeed risky, it finds its justification in biblical tradition and has therapeutic value.

Paul’s recommendations apply to Christians, but at the same time, they have a rather universal dimension. Even people who do not identify with any religious denomination may benefit from Paul’s therapeutic hints about the emotion of joy. After all, most humanity is ultimately engaged in achieving a certain well-being of the spirit, without which no development is possible.

For the exegetical analysis, I will use the historical critical method with special attention given to the Traditional Literary Criticism and Form Criticism. The main goal is to understand the analyzed text’s meaning in its historical context. In order to gain an even broader perspective, a synchronic reading will also be applied (Fitzmyer, 1995). In this regard, the canonical approach will be embraced showing especially how the text examined is related to other biblical passages where the motif of joy occurs.

### Emotion or *Páthos*?

I will refer to the joy postulated by the Apostle in 1 Thess. 5:16 in terms of emotions. A few caveats, however, must be made regarding the term “emotion.” Paul and the world of his contemporaries had not heard of “emotion” at all, as the term has been in use for only the last 200 years. Rather, the ancients used the term *páthos* (pl., *pathe*). In modern literature, the words *páthos* and emotion are often treated interchangeably, although *páthos* had a much broader meaning in Paul’s time. It embraced a broad spectrum of affective phenomena. It referred to all the adventitious changes that happened to a person, as opposed to actions that he consciously undertook. The word *páthos*, depending on the context, could describe, for example: feelings, affects, experiences, sensations, desires, or qualities. At the same time, both Plato and Aristotle also used it in a sense narrowed to affective phenomena, which we twenty-first century contemporaries have in mind when we speak of emotions. The problem here, however, is that Plato and Aristotle used it with a great deal of arbitrariness and inconsistency. It was not until the Stoics that the ancients developed a consistent theory of *pathe*. However, both the Stoics and the aforementioned classics of Greek philosophy believed that *pathe* were harmful and therefore, should all be banished (Sihvola & Engberg-Pedersen, 1998).

In this presentation, I will use the term “emotion” when referring to the joy postulated in 1 Thess. 5:16, knowing that it is not perfect in this regard. I take this liberty for the sake of the reader who is more familiar with this terminology. That said, it should still be noted that the concept of emotions is a phenomenon that remains difficult to grasp. We all experience them, but a problem arises when they need to be defined (Fehr & Russell, 1984). There are dozens of different scientific definitions depending on what field the researchers come from (Kleinginna Jr. & Kleinginna, 1981). Providing some new definition is beyond the scope of this study. What I am particularly interested in here is the relationship of emotions to reason and thinking.

Rosaldo (1984, p. 143) stated that “emotions are thoughts somehow felt in ‘flushes’, pulses, ‘movements’, of our livers, minds, hearts, stomachs, and skin. They are *embodied thoughts*, and thoughts seeped with the apprehension that ‘I am involved’.” Wierzbicka considers this approach rather unsatisfactory pointing out the fact that (1999, p. 2), “the English word emotion combines in its meaning a reference to ‘feeling’, a reference to ‘thinking’, and a reference to a person’s body. […] it requires a combination of all three elements (thoughts, feelings, and bodily events/processes).” Roberts (2008, p. 491) also notes that:

Paradigmatically, emotions are responses to situations of determinate types–for example, situations of threat, of offense, of good fortune, of aid, of rivalry, of loss, of good prospects, and so on. But the situations that human emotions are “about” are not usually simple stimuli […]; we respond to situations via one or another interpretive grid, so it is possible for a person who is in a situation of good fortune, say, to respond emotionally as though the situation were one of bad fortune, and vice versa. Often the interpretive grid with which we view a situation emotionally is a narrative or incipient narrative of our awareness of a situation’s historical background.

Thus, many human states or emotional dispositions are environmentally and culturally conditioned. Since these are susceptible to change, emotions—including joy—can also be shaped along with them (Morris & Maisto, 2008). In a different environment and culture, different emotions will be born, but also different stimuli will trigger them. It is therefore safe to assume that our working on emotions is possible. They can be consciously aroused, they can be tamed, or they can simply be worked out in a different manner. I believe this conviction guided Paul when he called for joy in his First Letter to the Thessalonians. So, it is worth first asking the question about the environment in which Paul lived and acted.

### St. Paul’s Background

Paul of Tarsus grew up on the border of two worlds: the Jewish and the pagan (Greek-Roman). On the one hand, his identity was shaped by Judaism and the texts of the Old Testament. But on the other hand, he was a Roman citizen coming from Tarsus. It is natural to think then that Greco-Roman culture had some impact on him (Ferguson, 1993; Hays, 2011). The two different mindsets, styles of thinking about man and the world around him, must have also affected his approach to emotions. So, it is worth at least briefly to outline the key differences in approaches to this phenomenon.

### Emotions in the Old Testament

The term emotion does not occur in Biblical Hebrew. However, the authors of the Hebrew Bible often describe in their own way what we would call emotional states. One gets the impression that emotions are an integral part of a believer’s life and identity (Kruger, 2015). This can be seen in the Psalter—a kind of prayer book of Israel. It can be said that the psalms serve as an “outpouring” of emotions before God (Iwański, 2015). They include prayers of praise—full of joyful excitement and even euphoria (e.g., Ps. 34, 92, 117)—as well as lamentations, that is, prayers for times of pain, suffering, and trepidation, which help to name and eject negative emotions (e.g., Ps. 3, 13, 30) (Polischuk & Kang, 2020).

It can be said with certainty that joy is at the very center of Biblical Judaism. God is to be served in a spirit of joy and celebration (e.g., Deut. 16:14–15). Religious holidays were established as a time of emotional renewal for Israel (Pawlowski, 2019). A great example of this is the description of the scribe Ezra’s public reading of the Law of Moses. The entire people gathered in Jerusalem and demanded that the words of the Law be read to them, which Ezra promptly arranged. After they were read, everyone burst into tears, realizing how far they had strayed from the standards contained in the Law of Moses. They all felt a tremendous sadness, but neither Ezra, Nehemiah nor the Levites instructing the people allowed them to weep and become downhearted. Instead, they commanded (!) the people to celebrate in an atmosphere of joy, and this indeed happened. The biblical author noted that there was a radical change in emotional state among those present: from sadness to joy (Neh. 8:9–12).

This scene also has symbolic value, since in Biblical Judaism, the experience of the liturgy is never dispassionate, but full of emotion. Periods of penitential sorrow are followed by periods of joyful celebration, as God comes to heal and forgive His people. Such celebrations are to be full of physical involvement of the participants—clapping of hands, loud and joyful singing, shouting (e.g., Ps. 37:11, Ps. 47:2.7), dancing accompanied by music (e.g., 2 Sam. 6:5.14–15), and offering sacrifices (e.g., Ps. 116:17). All this involves the whole person—including his emotions (Franck, 2021).

Anderson (1991, p. 13) notes that there is a sharp contrast between the modern and biblical idea of emotions. He notes that in both Biblical Hebrew and other Semitic languages, concepts such as “joy” often presuppose specific activities and actions. In detail, they can refer to the activities of eating and drinking (Ex. 32:6, Deut. 12:7, Neh. 8:12), donning festive garments (Ps. 30:12), singing (Ps. 137:3, 2 Chron. 23:18), and dancing (Ex. 32:19, Lam. 5:15), as well as anointing with oil (Ps. 45:8, Isa. 61:3) and offering sacrifices (Deut. 27:7). In this view, the modern account of emotions ignores or radically minimizes their behavioral dimension.

### Emotions in Greek Philosophy

The classics of Greek philosophy, such as Plato (†347 a.Ch.n.), Aristotle (†322 a.Ch.n.), as well as Zenon of Kition—the founder of Stoicism (†263 a.Ch.n.)—and Epicurus (†270 a.Ch.n.), although living long before the New Testament period, had a great influence on the mentality of the people of the New Testament era. They all had in common a great aversion to emotions (*pathe*), which they believed posed a great danger. Philosophy was to help them form the right beliefs to guard against such dangers (Konstan, 2006; Anderson, 1991).

It is worth recalling a somewhat amusing story from Plato’s life, which says a lot about his attitude regarding the emotion of joy. In the so-called Third Epistle, which was addressed to Dionysius II, the ruler of Syracuse, Plato refers to events at Delphi, where the addressee of the letter was said, in a fit of spontaneity, to have publicly wished the local god joy. Plato expresses his distaste for this behavior saying that he himself would not even wish this to a man, let alone to a god. Neither gods nor humans should be greeted with a call to rejoice, since in the case of the former, it is a wish contrary to their nature, and in the case of humans, it leads to indulgence toward each other, which results in thoughtlessness and exposes their souls to danger (Starr, 2013, p. 537).

In this context, it is still worth mentioning the Stoics, who developed the most consistent theory of emotions (*pathe*). The extent of its social influence could not be overestimated in the New Testament era (Łapiński, 2015). The Stoics^1^ believed that reason occupied a fundamental place in the human soul, and emotions (*pathe*) were inseparable from it. They distinguished four main emotions: sorrow (*lupe*), fear (*phobos*), desire (*epithumia*), and pleasure (*hedone*) (Banicki, 2006). Unfortunately, they did not consider them allies of reason. They rather saw them as diseases of the soul, from which only the influence of cool reason could cure, ultimately leading to peace of mind, that is, *apatheia* (Wigura, 2017). The Stoics not only ruled out uncontrollability (*akrasia*), that is, succumbing to emotions (*pathe*), but recommended the complete eradication of them (emotions) until *apatheia* was achieved (Plamper, 2015). A sage is a person free from *pathe*. He is emotion-free, that is, *apathes*. He is someone who has freed himself from fear, despair, pity, hope, jealousy, passionate love, intense joy, and many of their derivatives (Nussbaum, 1994).

During the so-called Late Stoic period, the Stoics developed the concept of *eupatheia*, or “good emotions.” They listed among them joy (*chara*), caution or discretion (*eulabeia*), wishing or willing (*boulesis*) (Inselmann, 2016). Of course, *chara* is the most interesting from the perspective of our study. That joy is defined as “rational uplift” (*eulogos eparsis*). Stoics were opting for joy “without enervating uncertainty, joy without fear and grief, a joy that really does move and lift up the heart.” At the same time, that joy, they argued, “has no commerce with laughter and elation.” It was rather “a stern matter” (*res severa*), as defined by Seneca (Nussbaum, 1994, pp. 399–400; Moors, 2010).

Summarizing this part of the argument, it should first be noted that both ancient and modern authors share the opinion that we are not powerless over emotions. We can avoid them, or distance ourselves from them (Stoics), or we can shape them in ourselves, and even change them on demand (biblical authors). The difference, however, is fundamental, for while the Stoics saw no possibility of achieving happiness (*eudaimonia*) without banishing all emotions, the biblical authors saw no chance of attaining spiritual and psychological well-being without encouraging emotions.

St. Paul, like the authors of the Old Testament, saw man holistically—including his emotions. Emotions were something positive and desired. Paul was aware that the Thessalonians had grown up in a cultural code that was quite different from such a mentality (Barton, 2013; Roszak, 2021). So, how could he convince them of a new way of thinking about emotions? Both the events of their own experience and the change in attitude (thinking) within the community would prove helpful. So, let us take a look at how he proceeded to convince them.

### Shaping Joy of the Thessalonians

When Paul wrote his first letter to the church in Thessalonica, the community there may have had a few dozen or, at most, a few hundred members. All of them probably experienced much spiritual “turbulence” following conversion, such as abandonment of old beliefs and adoption of new ones, as well as entry into a new community that was unprecedented. Even if it is true that the Thessalonians were generally a very practical people—for it was a merchant city—it is difficult to think that they remained untouched by all these new circumstances of life (Bruce, 1982; Elliott, 2005; Barton, 2011).

An allusion to this turmoil is found early in the Letter, where Paul writes: “And you, having received the word in the midst of great tribulation, with the joy of the Holy Spirit, became imitators of ourselves and of the Lord” (1 Thess. 1:6). For many commentators, the aforementioned “tribulation” (*thlipsis*) is some unspecified reference to the adversity that befell those in Thessalonica who opened themselves to the Gospel (Chapa, 1994). This supposition, moreover, is confirmed in 1 Thess. 2:14, where Paul refers to the suffering they experienced from their countrymen. The Thessalonians had ample reason to be stressed as victims of social ostracism and harassment from fellow residents and former friends (1 Thess. 2:14, 3:2–3). It is also possible that this is a reference to the spiritual struggles of the converts. So, it would be an echo of the emotional breakdown they experienced after they broke with the past to embrace the Gospel (Malherbe, 1987). It was a time of crisis in which their new identity was beginning to take shape (Horrell, 2002; Lieu, 2004).

But perhaps most acute was the suffering, the Thessalonians suffered as a result of the death of some respected members of the community (1 Thess. 4:9–10). This loss was a blow to the very heart of their still-fragile faith-based largely on hopes related to the Parousia—the Lord’s second coming (1 Thess. 1:10, 2:19, 3:13, 5:1–11) (Iovino, 1992; Nicholl, 2004; Barton, 2011). In the face of the addressees’ sorrow and grief (1 Thess. 4:13–18), “Paul offers comfort to the Thessalonians who were grieving. He begins with their grief for those who had fallen asleep (v. 13), then uses various traditions to provide the reason (v. 14.15) why they should refrain from grieving, and on that basis directs them to comfort one another (v. 18)” (Mahlerbe, 1983, p. 256). This positioning of the matter places Paul among the participants in the lively debate that was taking place in philosophical circles at the time. At issue, it was the question of whether sorrow and its externalization in mourning practices an appropriate response to death on the part of those who considered themselves truly wise (Konstan, 2016; Hope, 2017). Without a doubt, Paul did not mean to engage in a philosophical debate, but rather aimed at providing explanations that might have a therapeutic value. His purpose was not only to change the state of knowledge of the addressees, but to improve the state of their spirit by changing their attitude. If it were only about knowledge, the expected improvement might not occur. That’s why Paul explicitly demands the addressees’ involvement on the emotional level: rejoice always!

## Parenesis—A Therapeutic Rhetoric

The last two chapters of 1 Thess. are maintained in the genre called parenesis (May, 1999). This is a well-known form of speech in antiquity used by speakers who were concerned with shaping ethical attitudes. It was used to reassure others that the path taken was the right one, or to suggest that certain corrections be made. At the core, the parenesis is the presumption of a friendly bond between the author and his addressees. Often these ties are described in terms of family relationships—father and children (Malherbe, 1986). A parenesis is a condensed statement. More than irrefutable arguments, it provides short motivating explanations and uplifting encouragement. Not surprisingly, it is also used by Paul in this letter as a suitable means for his “rejoice therapy.” This form of communication is perfectly able to appeal to the emotions of the audience—so as to cheer them up and motivate them to take concrete action. This way the shaping of joy may well be accomplished.

For the sake of completeness of the picture, it should also be noted that in the verses of 1 Thess. 5:16–22 is also present a rhetorical formula identified as perora (Latin: *peroratio*) (Klauck, 2006). The perora is intended to appeal to the emotions of the addressees, making it possible to involve them in the content being conveyed. According to Aristotle, it was also intended to refresh the memory and influence the emotions of listeners/readers, as well as to reinforce the message regarding key issues (Witherington III, 2006). The purpose of the perora is to send one last message that is as gripping and uplifting as possible.

The verse about joy that interests us is precisely in the “heart” of the pericope, which has a concise five-part structure (12–13, 14–15, 16–18, 19–20, 21–22). Its “heart” is the verses of the third section (5:16–18). This is also where our verse on joy (5:16) fits in. To understand the joy postulated there, it is necessary to see it in the context of the other elements of this pericope.

### Section I. 1 Thess. 5:12–13: Mutual Respect

In this section, Paul seems to be turning his readers’ attention away from the dead and toward the living. There, he demands respect for those who “toil” in the community. Respect for them and their work protects against entitlement. The latter kills joy and makes unbearable both the life of the person concerned, as well as those living around him. Only by getting rid of entitlement does a person become capable of building good relationships. In our context, it is about community relations, which Paul shows by comparing it to family life. An attitude of respect also makes peaceful coexistence within the community possible. Therefore, section 5:12–13 concludes with the words *eireneute en heautois* “keep peace among yourselves.” A community where there is peace is much more eager to rejoice (Thompson, 2011).

### Section II. 1 Thess. 5:14–15: Empathy and Responsibility

Proper respect for those in charge of the community gives rise to the need for personal involvement in community affairs. There are weak, depressed, internally broken people who have a right to be there too. Unfortunately, they can turn out to be toxic. Instead of looking on passively, one must instruct and correct, but by no means repay evil for evil. The desire to retaliate is equally toxic. Any injustice about which we nurture a desire for retribution by no means ennobles a person but in fact impoverishes a person. Thus, a Christian who engages his emotions on the side of revenge against anyone enters the path of self-destruction. In him, joy has no chance to blossom.

As an antidote, Paul recommends to the Christians in Thessalonica to “chase” the good. The idea is that they should not wait until an opportunity to give good presents itself, which they then graciously seize. They are to create such opportunities themselves. In this way, their thinking will change. Instead of getting wrapped up in how to repay an injustice, they are to get involved on the side of good.

### Section III. 1 Thess. 5:16–18: Joy, Prayer, and Gratitude

Finally, in verse 16, the key words are said: “Rejoice always!” The apostle takes the content given here very seriously. He not so much recommends but commands the Thessalonians to always be joyful.

*Pantote hajrete*, “Rejoice always!” (5:16). The adverb *pantote*, “always,” also appeared in the previous verse, where there was talk of chasing after good things. The author wants to show that joy is also something to be sought. It is something fleeting that cannot be acquired once and for all. Paul is aware that also inherent in every emotion—including joy—is impermanence. That’s why, once its “supply” runs out, one must strive for a new one. “Always” means again and again and then, again. One must constantly strive for it.

Paul, demanding joy from the addressees, reminds us of Ezra, who commanded the weeping Israelites to change their state of mind. Yet, the Apostle’s command was an unusual one, given the background of the culture and mentality of the time. After all, the aforementioned Plato or the Stoics considered emotions to be diseases of the soul, which had to be eradicated from life. Freeing oneself from them was tantamount to achieving peace of mind, called *apatheia*. Joy, if it already arose, was supposed to be an “indifferent” thing for the Stoic (White, 2006).

Meanwhile, Paul makes it unequivocally clear that joy is something desirable. Like peace, it is a gift of the Holy Spirit (Gal. 5:22), which has already been granted to them. Faith and a new Christian identity directly must give rise to joy. On the other hand, if joy is lacking, it is a signal that something is missing in the realm of faith and identity. Therefore, not only should it be wished for, but actively sought. Joy is also to be outwardly visible as a kind of “trademark” of Christians.

It seems crucial to understanding the meaning of 5:16 to see it as a kind of culmination of the words already spoken at the beginning of the Letter in 1:6. There, the theme of joy first appeared. Paul recalled the enthusiasm and joy with which the Thessalonians had accepted the Gospel despite suffering. He especially emphasizes that although the bad experiences did not end for the Thessalonians, they were still able to feel the joy that came directly from the Holy Spirit. In other words, he is recalling here their own experience, when joy was granted to them; even though they were hardly in a disposition for joy as a result of the suffering, they were experiencing. The present situation of the Thessalonians, to which Paul refers in chapters 4 and 5, is somewhat analogous to that of the early church in Thessalonica. The Thessalonians originally opened themselves to the joy coming from the Holy Spirit, despite circumstances unfavorable to joy. They could do it back then, they would be able to now as well.

But to call on the grieving people to show joy instead of licking their proverbial wounds seems at first somewhat inhuman. Paul, however, is far from downplaying human suffering. He merely wants to reach his addressees with a message: suffering does not exclude joy. It is possible to suffer for the rest of one’s life because of some loss, mistakes made, or experiences, and yet remain a joyful person. So, now it is only up to the Thessalonians whether they will increase the ranks of the “tearful” and depressed who cannot be consoled, or whether they will remember the recent phenomenon when they allowed the Holy Spirit to work and once again experience the “impossible”—joy despite suffering.

It cannot be unnoticed that in Paul’s view joy is part of God’s plan for us (5:18). Since this is the case, after all, God could not possibly want something impossible for us, could He? It seems that it is with such confidence that Paul can recommend remaining in lasting joy that he knows half the work (if it can be measured at all) has been done. God has already equipped the Thessalonians with what it takes to be joyful. One might say that “the ground has been prepared.” In other words, it is a matter of grace—a gift given freely by the Holy Spirit. However, man must do his part for joy to shine in him. Paul reminds the Thessalonians of this important fact.

#### 1 Thess. 5:17: “Pray Continually”

The apostle repeatedly mentioned his constant prayers (cf. 1 Thess. 1:2–3, 2:13, 3:10, see also Phil. 1:9, Rom. 1:10, Philem. 4). However, someone might wonder: “is it possible to pray constantly? Even if I wanted to, I am physically unable to do it continuously!” Moreover, the Church in Thessalonica is in *statu nascendi*. The Christians there do not have some elaborate palette of prayers from which to draw. Even if one were to assume that Paul presented a treasure trove of prayers such as the Psalter, he at the same time assigned them quite a few tasks—instruct, comfort, admonish, and chase after the good. Their implementation required considerable time. Besides, everyone still had to earn a living somehow. So, is Paul contradicting himself now? Not at all! For him, prayer simply does not end with the recitation of a psalm or other devotional text.

The key to understanding this conundrum is found in his words in 1 Cor. 10:31: “Therefore, whether you eat or drink, or whatever else you do, do everything to the glory of God!” In other words, every activity, even the least appreciated, should be dedicated to God. Then, it becomes a prayer! See what this could look like in practice: You get up in the morning and dedicate all the activities of the day to God in a quiet chat with Him. Everything you undertake is immediately dedicated to God. When you choose to glorify God through every activity, everything begins to shimmer in completely different colors. First, you will think ten times before you decide to do or say something negative or unkind, because you feel that it will not be an activity for God’s glory. In contrast, any good that comes through your hands or mouth will glorify God and come back to you. Paul does not change the meaning of traditional prayer, but he insists on seeing it in a broader perspective. Only then can it positively affect spiritual well-being and bring the expected joy.

It is still worth looking at the references to the prayers of Paul himself that occur in the First Epistle to the Thessalonians. First are the prayers of thanksgiving. Early on, Paul mentions that he and his associates “always” (!) give thanks to God for the Thessalonians in their prayers (1:2; cf. also 2:13, 3:9). The latter does not mean that Paul was in a state of constant prayerful exultation, only that he always mentioned them in his daily prayers (Longenecker, 2002). In 1 Thess. 3:10, he says that he prays fervently day and night—that is, always. Does this mean that he literally does nothing else? No! If that were the case, then, the Epistle in which these words were included would not have been written.

#### 1 Thess. 5:18: “Be Thankful”

*En panti eucharisteite*— “In everything (in every situation) give thanks.” Is it possible to be thankful when we face hardships, chicanery, or tragedies? Speaking to the Thessalonians about joy, as well as an attitude of thanksgiving in every situation, may have been irritating to their ears. Yet Paul, after all, did not live in ignorance of the circumstances of their lives. Nor was he in the habit of idealizing and imposing impossible expectations on people, or things that he himself had not lived. It seems that Paul shows some amazing intuition here, making his point that an attitude of gratitude can further help achieve a joyful disposition.

This nuance already appeared in the first section of the pericope in question, when respect for leaders and their work was mentioned (vv. 12–13). What else was this respect a result of, if not gratitude? Let no one think that he is entitled to something! Such an attitude leads to pathology and, in fact, to a kind of cynicism, from which joy certainly does not come. Instead, let us know how to accept with gratitude what others do for us. From this gratitude, it becomes joy and the need for a certain symmetry on the part of the recipient. I get something, I am being served, but I do not get used to it as if it is due to me. If the gifting stops for some reason, I will retain a good memory of the good I received. Otherwise, I will grumble, kick, and bite, because something to which I allegedly had an inalienable right was taken away from me.

We are commanded to be thankful. On the one hand, in any situation, you must find enough reasons to give thanks for the graces you have already received. The recollection of what you have been blessed with pours new hope, gives wings, and thus actively contributes to sustaining joy. On the other hand, the exhortation to be thankful comes immediately after the encouragement to pray unceasingly. It is also known that a person who knows how to give thanks and show gratitude and appreciation inspires everyone around him. All of this together—joy, prayer, thanksgiving (gratefulness)—is something that is in line with God’s will when it comes to the profile of a Christian. Christianity is a bumpy but joyful path in the presence of God, who wants man to be complete, joyful, and fulfilled.

### Section IV. 1 Thess. 5:19–20: “The Spirit do not Extinguish! Prophecy do not Disregard!”

“The Spirit do not extinguish!” The Thessalonians came from among people with a certain work ethos. Their city was located on a busy trade route. Living in this commerce, they themselves were also probably a very rational and strong-minded people. The charisms, the gifts of the Holy Spirit that manifested themselves in their gatherings, may have aroused skepticism. Therefore, Paul encourages them not to underestimate the charismatic dimension of their community’s life. The Holy Spirit is not meant to enslave people, but precisely to open and “shower” them with various gifts—including the gift of joy. In this action, the Holy Spirit is powerfully effective, but also subtle. A Christian can put up a barrier against it—close himself to it or, in the words of Paul, extinguish it. The latter is an appropriate warning with reference to the Holy Spirit, who is associated with fire (e.g., Isa. 30:27–28, Acts 2:3–4). The Holy Spirit can do nothing in a person without his consent. So, if these gifts remain at hand, and yet are unused, then, an unimaginable waste takes place.

Paul is aware that without the Holy Spirit there is no prophecy. According to him, this gift was of immeasurable importance for the development and survival of the community. Through prophecy, the community was able to build itself up, enrich itself, and receive the right encouragement for action and development. Contrary to popular belief, a prophet is not one who foretells the future. In the biblical view, the prophet rather is one who is focused on the present. He is supposed to interpret the present time in light of God’s will. Ultimately, it is about embodying God’s will, and accepting His will and plan for each of us. When we attempt to live our lives in this way, our spiritual well-being is strengthened and fortified. This, in turn, makes us capable of living in joy.

### Section V. 1 Thess. 5:21–22: “Examine Everything and Hold on to What is Good! From Everything that has the form of Evil, Stay Away”

The call to show a healthy criticism of everything is also extremely bold. It is about discerning everything that a Christian encounters daily. The first criterion for discerning what is good is always God’s will for man (Rom. 12:2) (Munzinger, 2007). As I mentioned earlier, this is not synonymous with forbidding or restricting, but with guiding the Christian in such a way that he is free, happy, and “made complete.”

The call to discernment, in a way, encourages Christians to actively participate in the life of the so-called world. It protects against seeing the community as a hermetic group, a “besieged fortress.” There is good outside, too. Even non-believers are inclined to goodness and do good. Your task as a Christian is to evaluate everything, examine it and decide whether you are dealing with good or evil. You have enough discernment to know already whether something is good or not. Do not be afraid to learn about the world. A Christian is an enlightened person who is not afraid of the world but is in dialogue with it. However, he is not a mindless consumer, but is able to choose consciously. So, Paul continues: “avoid everything that is evil!” And here again the Apostle leaves a large space for the freedom of the Thessalonians. If something has even a semblance of evil, one should stay away from it. Not just for ethical reasons, but pragmatic ones. Simply put, evil harms us. Consequently, no real joy will come out of it.

## Discussion

St. Paul, in his first letter as we know it, attempts to embody the biblical practice of changing negative emotions to positive ones—that is, joy—despite objective difficulties. He does this to the members of the Thessalonica church community, who had grown up with the standards appropriate to the Greek philosophical schools. He urges the addressees to rejoice always. Paul’s treatments could remind the Thessalonians of the therapeutic exhortations of the Stoics who preached dispassion in the face of suffering. However, Paul’s treatments were fundamentally different from Stoic practices. While the Stoics would have recommended getting rid of any emotion to pave the way for reason, Paul sees no contradiction between emotion and reason. The knowledge that reason will take possession of us is understood, but it still has no therapeutic value. Only emotional—joyful—involvement on the part of Paul’s readers guarantees therapeutic success.

Paul places joy, understood in this way, at the core of the new identity that Christians should develop. It is, as it were, their “trademark.” This is so important that when a Christian lacks joy, the question should be asked whether he is unsure of his Christian identity. The Holy Spirit enables our Christian hearts to accept all his gifts, including the gift of joy. It is God’s will for man to be joyful. Sadness is not evangelical. So, there is no reason for us to cherish it.

In 1, Thessalonians Paul employed more than just rhetorical strategies to achieve the intended effect of his “Rejoice Therapy.” He provided his readers with practical and universalistic techniques, if you will, especially these three which follow here:

### Calling to Mind Positive Memories

The issue of joy appears at the beginning and end of the First Epistle to the Thessalonians. This is no coincidence. First, Paul recalls their shared experience from the past. The Thessalonians received the Gospel amid great tribulation, but this did not prevent them from experiencing the joy that flows from the Holy Spirit (1 Thess. 1:6). So, it is possible to be joyful even though circumstances do not fill one with joy. This is the personal experience of the Thessalonians themselves. That is why, in the conclusion, Paul does not sound like a theoretician, for whom joy is the subject of some abstract, academic considerations (1 Thess. 5:16). He is referring to pure facts which accompanied their birth anew as Christians in their hometown. He purposefully recalls those astonishing moments when the impossible—joy amidst tribulation—became a reality. Paul wants his addressees to believe that with a little faith on their part, they can see this transformation happen again. The memory of it all could prove invaluable for changing their state of mind for the better.

It seems that all of us have similar experiences with memories. It may prove very therapeutic in times of crisis to think of the people, moments, and places; we treasure as uplifting and particularly enriching. A happy childhood can be a good example here. We like to think back to the people who surrounded us with warmth, even if there was poverty at home. Relationships with loved ones have proved much more important in our lives than difficult circumstances. We like to return with our thoughts to such places and moments when we felt carefree and joyful. If, in addition, we have the good fortune to actually return to the places or environment where good memories originated, we can expect a kind of spiritual “rejuvenation.” Just stepping over the threshold of the family home, where we were loved, is enough for us to be flooded with good feelings, and thanks to which we can really relax. Even if some of those loved ones who provided us with a carefree adolescence are no longer there—our grandmothers, grandfathers, parents—time seems to stop in such places. This immediately has an uplifting and, up to a point, therapeutical effect. The same effect can even be achieved sometimes by looking through old photographs of family and friends. Perhaps that is why, intuitively, many of us like to surround ourselves with photos that recall good people and happy moments that we experienced. These simple things can bring quite a bit of joy.

### Treasure and Protect that “Pearl!”

By calling on the Thessalonians to rejoice always, Paul seems to be saying: your joy must not depend on circumstances. Of course, Paul is aware that joy—like other emotions—is susceptible to transience, but it is also, unfortunately, often subject to “theft.” He seems to present a simple logic about this when reminding the Thessalonians to be joyful. To paraphrase him: “There are many things and people who will do everything to take away your joy. However, they are powerless unless you allow them power. No one can take your joy away from you if you do not give it away yourselves. You should look at joy as a priceless evangelical pearl. As such, it must be kept as you would treasure, safe from damage by intruders. Instead, protect that ‘pearl’ and make good use of it.”

### “Converting” Thinking

In general, the Apostle’s “rejoice therapy” aims at “converting” the Thessalonians’ thinking. Ultimately, thinking is the activity that is first in line to be “healed.” Paul’s approach is complex but clear. He lays it out in the whole pericope in 1 Thess. 5:12–22. The call to rejoice is not really feasible without a change in thinking and attitude regarding issues of relationship. Here is how an alternative version of Paul’s talk might sound: “Stop focusing so much on yourselves. Do not assume that you are entitled to anything. Instead of taking things for granted, have gratitude. Give thanks even in a difficult situation. Instead of classic complaining, act! Show empathy and sensitivity. Repair relationships with those with whom you can. Pray always—both with words to glorify God and by suffusing every moment with prayer. Let every activity be dedicated to Him. You will see how your environment and those in it will then change. In difficult moments, do not be seduced by the temptation to lament that nothing ever works out for you in life and that you never got anything from anyone, because this kills joy. When something unpleasant happens, it is easy to forget past happy days or years. Just think back for a moment to all the good things you experienced. You will never run out of pretexts for sadness, but there are always more reasons to be happy.”

The study of emotions (passions) in the Bible has been gaining growing attention among biblical scholars. However, there are many challenges to be faced as in every pioneer work. The most important one is the fact that no methodology has yet been established in this regard—as Inselman observed (2016, p. 537). So, one must carefully apply the so-far-accepted methods with a creative mind trying to incorporate factors that may help with studying emotions belonging to a different historical and cultural environment.

### Limitations

I am aware that the analyses presented here may run the risk of being somewhat anachronistic. So does every contemporary approach concerning ancient matters. Up to a point, it is always speculative since we must rely on sources that give us only a limited picture of the ancient world and ancient mindset (Van Cappellen, 2017). On the other hand, we may not be shy about studying these texts in hope that they will resonate with us contemporary readers (Oviedo, 2022).

## Conclusion

The aspect of shaping joy found in 1 Thess. seemed especially relevant for the post-pandemic day and age we live in. I believe that a more psychological approach to the text studied might bring to light some additional aspects how to shape and keep personal joy. Inselman (2016, p. 545) rightly noted that “emotions and passions are always embedded in psychological processes.” It would take an expert in psychology to address some questions regarding joy as presented in 1 Thess.

Finally, it is worth mentioning that counseling centers based on “joy therapy” have recently sprung up. One of them is the Rejoice Counseling Apostolate (rejoicecounseling.com), which seeks to “journey with you to joy” for people who are struggling with life’s difficulties. Another is Catholic Counselors (catholiccounselors.com) which uses 1 Thess. in its online advice on “Cultivating Joy” (March 8, 2014). Edward Hays', 2007 popular book "Chasing Joy: Musings on Life in a Bittersweet World" is also worth mentioning in this context. His book was referenced in the Oct. 3, 2011, issue of “America,” in the article “Rejoice Always: The Surprising Joyful Theology of 1 Thess,” which brought this theme to a large readership.

## Footnotes

1 Stoicism was not a monolith. As a rule, three phases in its development are distinguished. The one in which St. Paul lived constitutes the last of the phases. This period is referred to as the “Late Stoa.” In analyzing the views of the Stoics on various issues, one would have to assume an evolution of thought. Researchers often come across discussions of the representatives of this movement themselves, in which they represent quite different views on some topics. The topic of emotions (*pathe*) is no exception. However, the detailed survey of the views of individual representatives of this trend, considering different schools they were representing, makes each such a presentation somewhat unbearable for the average reader. Therefore, following the example of Miech (2009, p. 138), I was tempted to briefly present the issue in question, treating Stoicism as a unity. I am aware that experts in the field may find this approach anachronistic, but I do so to facilitate reception of the presented content by readers who do not have the competence proper to experts.
